# Dedicator of Cytokinesis 4: A Potential Prognostic and Predictive Biomarker Within the Metastatic Spread of Breast Cancer to Bone

**DOI:** 10.1177/1176935119866842

**Published:** 2019-08-26

**Authors:** Janet E Brown, Jules A Westbrook, Steven L Wood

**Affiliations:** Department of Oncology & Metabolism, Academic Unit of Clinical Oncology, The University of Sheffield, Sheffield, UK

**Keywords:** Bone metastasis, breast cancer, proteomics, predictive biomarker, cell motility, bisphosphonate treatment

## Abstract

Metastasis to bone occurs in over 70% of patients with advanced breast cancer resulting in skeletal complications, including pathological fractures, hypercalcaemia, and bone pain. Significant advances have been made in the treatment of bone metastases, including the use of antiresorptive drugs, such as bisphosphonates, as well as antibody-based therapies targeting key signalling intermediates within the process of cancer-mediated bone destruction. Despite these advances, treatment is not without side effects, including osteonecrosis of the jaw therefore biomarkers predictive of which patients are at high risk of developing bone spread are required to enable personalized medicine initiatives within this important disease area. We used proteomic analysis to compare the protein expression within (1) a parental triple negative human breast cancer cell line, (2) a fully bone homing cell line and (3) a lung homing cell line. The bone and lung homing cell-lines were derived by intra-cardiac injection of fluorescently labelled cells within immune-compromised mice. Proteomics identified Dedicator of Cytokinesis 4 as a biomarker predictive of bone spread, and this finding was further supported by the observation that high levels of Dedicator of Cytokinesis 4 within primary breast tumours were predictive of breast cancer spread to bone. Here, we provide an overview of this study and put the findings into context.

**Comment on:** Westbrook JA, Wood SL, Cairns DA, et al. Identification and validation of DOCK4 as a potential biomarker for risk of bone metastasis development in patients with early breast cancer. *J Pathol*. 2019;247(3):381-391. doi:10.1002/path.5197. PubMed PMID: 30426503. https://www.ncbi.nlm.nih.gov/pubmed/30426503.

In our recent publication,^1^ we have described the discovery of the role of Dedicator of Cytokinesis 4 (DOCK4) as a potential biomarker protein predictive of bone metastasis within breast cancer. Bone is a common site of metastasis within advanced breast cancer, and over 70% of advanced breast cancer patients develop bone metastases.^2^ Patients present with symptoms of bone metastasis in many cases years after apparently successful treatment, with disseminated tumour cells remaining dormant within bone prior to their activation. The cellular mechanisms leading to both breast cancer metastasis to bone, and the eventual reactivation of dormant breast cancer cells, are currently the subject of intensive research. Bone is the subject of constant turnover with bone homeostasis being regulated by the activity of bone-resorbing osteoclasts and bone-forming osteoblasts.^3^ Disseminated and reactivated breast cancer cells secrete parathyroid hormone–related protein (PTHrP), which triggers osteoblasts to increase their synthesis and secretion of receptor activator of NF-κB ligand (RANKL), as well as reduce their secretion of osteoprotegerin (OPG) (an orphan receptor for RANKL). This results in increased binding of RANKL to the receptor for RANKL (RANK) resulting in osteoclast activation and bone destruction. Degradation of bone releases growth factors trapped in the bone matrix, which in turn stimulate breast cancer cell proliferation and release of PTHrP, resulting in a ‘vicious cycle’ of bone destruction.^4^

Current treatments for bone metastasis within breast cancer include bisphosphonate drugs (such as zoledronic acid)^5^ and antibody-based therapies, eg, denosumab.^6^ These treatments have greatly improved patient quality of life, with zoledronic acid conferring a statistically significant improvement in invasive disease–free survival within women who were >5 years post-menopausal.^7^ Despite these improvements in patient treatment, these drugs are not without side effects, including the case of bisphosphonate treatment of osteonecrosis of the jaw.^8^ To date, there are no effective biomarkers available to predict which patients are at greatest risk of developing bone spread, enabling a personalised medicine approach towards the treatment of skeletal metastasis within breast cancer.

We are engaged in research aiming to elucidate the molecular basis of cancer spread to the skeleton. This work has benefitted from access to unique patient sample banks, including samples from the large, open-label, international, multicentre, randomised, controlled, parallel-group phase 3 trial AZURE, which asks the question ‘Does adjuvant zoledronic acid reduce recurrence in patients with high risk localised breast cancer?’. The AZURE trial has provided both tissue samples (tissue microarrays) as well as patient serum and plasma samples from patients receiving standard adjuvant therapy either with or without addition of zoledronic acid. In addition, proteomic methods have been used to characterise murine models of breast cancer spread to bone, including intracardiac injection models of bone metastasis.^9^ Our proteomic analysis of bone-homing and parental triple negative breast cancer cells derived from intracardiac injection murine models has identified a panel of potential bone metastasis biomarker candidates including macrophage actin-capping protein (CapG), the PDZ-domain containing adaptor protein GIPC1,^10^ and the cell motility regulator DOCK4.^1^

DOCK4 was identified as a potential biomarker predicting bone metastasis risk via quantitative proteomic analysis of bone-homing and parental MDA-MB-231 cells using stable isotope labelling by amino acids in cell culture (SILAC).^1,11,12^ DOCK4 expression was elevated 2.5-fold within bone homing cells and was not elevated within a lung-homing control cell line, suggesting bone specificity. Immunohistochemical assessment of DOCK4 expression levels within AZURE-patient derived tissue microarrays (n = 689) demonstrated that DOCK4 levels were significantly prognostic for first recurrence within bone in control arm standard chemotherapy) patients (hazard ratio [HR]: 2.13, 95% confidence interval [CI[: 1.06-4.30, *P* = .034), an effect not observed within the zoledronate treatment arm (HR: 0.812, 95% CI: 0.176-3.76, *P* = .790).^1^ There was no association of DOCK4 levels with metastatic spread to nonbone sites suggesting that DOCK4 levels within primary breast tumours predict aggressive disease and risk of metastasis specifically to bone. The absence of a predictive effect within the zoledronate-treated arm suggests that DOCK4 levels may also inform patient treatment decisions.^1^ There was no statistically significant difference in overall survival between patients with high and low DOCK4 levels within this study.

Our study aimed primarily to look into the role of DOCK4 as a potential predictive marker for bone metastasis. Within our study, high DOCK4 levels reduced the incidence of nonskeletal metastases within patients in the control arm of the AZURE trial.^1^ This effect was not observed within the zoledronic acid treatment arm, suggesting that zoledronate may remove an advantageous effect of high DOCK4 levels upon the incidence of nonskeletal metastases. The mechanism of action of zoledronate on nonbone metastases is currently unknown; however, it has been previously described that zoledronic acid can inhibit the proliferation, survival, and migration of breast cancer cells.^13^ DOCK4 levels may therefore have prognostic potential for nonskeletal as well as skeletal metastases.

The role of DOCK4 as a prognostic biomarker for metastatic spread to bone was a unique finding within our published study. DOCK4 is a known guanine nucleotide exchange factor (GEF) responsible for activation of several GTPases inside the cell, including the GTPase protein Rac1, a pivotal regulator of cell migration expressed at the leading edge of motile cells.^14^ The mechanism of action of DOCK4 within the regulation of cell migration is still the subject of active research. Previous studies have demonstrated that the proangiogenic growth factor vascular endothelial growth factor (VEGF) promotes the interaction of DOCK4 with DOCK9 (a GEF for the GTPase Cdc42).^15^ In addition, VEGF-dependent Rac activation by DOCK4 is required for the activation of the GTPase Cdc42, and VEGF-dependent Rac activation is itself dependent on the activation of a further GTPase RhoG.^15^ In this way, the activity of a GTPase signalling cascade functions to both activate DOCK4 and transduce signals downstream of this important GEF. DOCK4 activation leads to the formation of lateral filopodia within epithelial cells within tumours, and the formation of blood vessel lumen within tumour angiogenesis.^15^ DOCK4 thus plays a pivotal role within tumour angiogenesis; however, its precise role within bone-specific metastasis is still to be elucidated.

While elucidating the role of DOCK4 within cancer spread to bone, we must be aware that proteins often play pleiotropic roles within the cell. Research within our group previously identified actin capping protein (CapG) as a prognostic biomarker for risk of breast cancer spread to bone. Actin-capping proteins do not appear to offer an obvious bone-specific function for metastatic spread; however, subsequent research revealed that CapG can act as an epigenetic regulator responsible for the increased expression of a prometastatic gene stanniocalcin-I (STC-I), required for metastasis to bone.^16^ Discovered biomarker proteins may thus achieve their effects via functions that differ from the primary gene annotation, and, thus, the pro–bone-metastatic role of DOCK4 may involve additional functions to the regulation of cell motility via GTPases.

The expression of DOCK4 is induced by the cytokine transforming growth factor-β (TGFβ), a pivotal cytokine within bone metastasis.^17^ TGFβ acts via Smad phosphorylation to increase the expression of numerous gene products, including c-MAF, a transcription factor responsible for promoting bone metastasis within breast cancer.^18^ Previous research within our group has also identified GIPC1 (a scaffolding protein and binding partner of the TGFβ receptor) as a potential biomarker prognostic for increased risk of bone metastasis within breast cancer.^10^ DOCK4 expression levels may thus be positively correlated with the expression levels of c-MAF,^19^ suggesting that, within bone metastasis of breast cancer, TGFβ increases expression of both c-MAF and DOCK4. DOCK4 may thus act as a functional mediator of a c-MAF response panel. The precise role of DOCK4 function within metastasis to bone is still to be elucidated. Studies conducted within our laboratory demonstrated that stable knockdown of DOCK4 expression reduced breast cancer cell motility; however, this effect was transient, being significant at 6 hours post knockdown, but not observed as significant levels 12 hours post knockdown.^1^ Cell motility may be the only aspect of DOCK4’s role that is altered in bone metastasis of breast cancer.

Our research to date has clearly identified a series of proteins with elevated expression specifically within bone metastasis of breast cancer. Functional study of these proteins has identified that they are frequently downstream of key cytokines and growth factors within skeletal metastasis, including TGFβ. We anticipate that DOCK4 will be just one protein within a panel of proteins ultimately forming a prognostic test for clinical application. Multiprotein tests have greater utility for informing patient treatment than single-protein tests,^20^ and have a greater ability to deal with diseases with known molecular heterogeneity, such as breast cancer. Research efforts are therefore currently underway to further supplement our protein panel with additional proteins, thus increasing the sensitivity and specificity of this clinical test when eventually applied. There are many aspects of DOCK4’s role within bone metastasis of breast cancer, which remain to be elucidated, including the possible role of DOCK4 within nonbone metastasis, as well as the interaction of DOCK4 with bisphosphonate treatment and its potential role as an informative biomarker for patient treatment decisions.
